# Predicting neurodevelopmental outcomes in extremely preterm neonates with low-grade germinal matrix-intraventricular hemorrhage using synthetic MRI

**DOI:** 10.3389/fnins.2024.1386340

**Published:** 2024-08-07

**Authors:** Chunxiang Zhang, Zitao Zhu, Kaiyu Wang, Linlin Wang, Jiaqi Lu, Lin Lu, Qingna Xing, Xueyuan Wang, Xiaoan Zhang, Xin Zhao

**Affiliations:** ^1^Department of Radiology, The Third Affiliated Hospital of Zhengzhou University, Zhengzhou, China; ^2^Henan International Joint Laboratory of Neuroimaging, Zhengzhou University, Zhengzhou, China; ^3^Medical School, Wuhan University, Wuhan, China; ^4^GE Healthcare, MR Research China, Beijing, China; ^5^Shulan (Hangzhou) Hospital Affiliated Hospital of Zhejiang Shuren University, Shulan International Medical College, Hangzhou, China; ^6^New York University, New York, NY, United States

**Keywords:** synthetic MRI, germinal matrix-intraventricular hemorrhage, extremely preterm infants, neurodevelopmental outcomes, predictive modeling

## Abstract

**Objectives:**

This study aims to assess the predictive capability of synthetic MRI in assessing neurodevelopmental outcomes for extremely preterm neonates with low-grade Germinal Matrix-Intraventricular Hemorrhage (GMH-IVH). The study also investigates the potential enhancement of predictive performance by combining relaxation times from different brain regions.

**Materials and methods:**

In this prospective study, 80 extremely preterm neonates with GMH-IVH underwent synthetic MRI around 38 weeks, between January 2020 and June 2022. Neurodevelopmental assessments at 18 months of corrected age categorized the infants into two groups: those without disability (*n* = 40) and those with disability (*n* = 40), with cognitive and motor outcome scores recorded. T_1_, T_2_ relaxation times, and Proton Density (PD) values were measured in different brain regions. Logistic regression analysis was utilized to correlate MRI values with neurodevelopmental outcome scores. Synthetic MRI metrics linked to disability were identified, and combined models with independent predictors were established. The predictability of synthetic MRI metrics in different brain regions and their combinations were evaluated and compared with internal validation using bootstrap resampling.

**Results:**

Elevated T_1_ and T_2_ relaxation times in the frontal white matter (FWM) and caudate were significantly associated with disability (*p* < 0.05). The T_1_-FWM, T_1_-Caudate, T_2_-FWM, and T_2_-Caudate models exhibited overall predictive performance with AUC values of 0.751, 0.695, 0.856, and 0.872, respectively. Combining these models into T_1_-FWM + T_1_-Caudate + T_2_-FWM + T_2_-Caudate resulted in an improved AUC of 0.955, surpassing individual models (*p* < 0.05). Bootstrap resampling confirmed the validity of the models.

**Conclusion:**

Synthetic MRI proves effective in early predicting adverse outcomes in extremely preterm infants with GMH-IVH. The combination of T_1_-FWM + T_1_-Caudate + T_2_-FWM + T_2_-Caudate further enhances predictive accuracy, offering valuable insights for early intervention strategies.

## 1 Introduction

Germinal matrix-intraventricular hemorrhage (GMH-IVH) poses a significant challenge in extremely preterm infants despite advancements in neonatal care. While survival rates for extremely preterm infants have increased, the incidence of GMH-IVH, particularly in its low-grade forms (Grade I and II), remains high, affecting around 44.68 percent of premature infants (Brouwer et al., 2014; Parodi et al., 2020). GMH-IVH continues to be a prevalent and clinically significant issue in preterm infants, especially those born extremely premature. The brains of extremely premature infants exhibit a higher germinal matrix volume, dense capillary networks, reduced support structures (Kidokoro et al., 2014), fewer anastomotic branches in the subependymal artery (Anstrom et al., 2002), oligodendrocytes are immature. Consequently, lower gestational ages render infants more vulnerable to the effects of cerebral blood perfusion and pressure, predisposing them to GMH-IVH (Gilard et al., 2020).GMH-IVH screening relies primarily on head ultrasound. Infants with low-grade GMH-IVH (grade I or II) are clearly at a much lower risk for developmental disabilities than infants with grade III GMH-IVH (Hambleton and Wigglesworth, 1976). Detecting microstructural damage to periventricular and subcortical white matter following low-grade GMH-IVH poses challenges; as such damage may be elusive. Furthermore, the reliability of head ultrasound in grading mild hemorrhages is limited, complicating the prediction of adverse outcomes (Leijser and de Vries, 2019). MR imaging is increasingly used in the extremely preterm population to complement cranial sonography to improve prognostic information and inform current clinical and future supportive care. It has been shown that low-grade GMH-IVH is followed by microstructural impairment, which are undetectable by ultrasound and conventional MRI. Consequently, there is a pressing need to identify new biomarkers for cerebral development in extremely preterm infants, offering potential for early detection and prevention of low-grade GMH-IVH.

Synthetic MRI, utilizing a multi-delay multi-echo sequence, emerges as a promising tool for simultaneously quantifying longitudinal relaxation time (T_1_), transverse relaxation time (T_2_), and proton density (PD). These parameters reflect microstructural changes during brain maturation, facilitating the objective assessment of abnormal maturation or disease conditions. Prior studies highlight the potential of T_1_ and T_2_ obtained from MRI at term-equivalent age (TEA) as imaging biomarkers for predicting neurodevelopmental outcomes in premature infants (Vanderhasselt et al., 2021; Dong et al., 2023). Synthetic MRI’s quantitative parameters effectively capture brain tissue characteristics and volume changes in premature infants with intraventricular hemorrhage, with T_1_, T_2_ relaxation times, and PD play crucial roles in diagnosing and evaluating intraventricular hemorrhage (Zhang et al., 2021). Synthetic MRI has shown promise in identifying early prognostic biomarkers for neurodevelopmental impairment, indicating its potential in predicting adverse outcomes linked to GMH-IVH (Vanderhasselt et al., 2021; Zhou et al., 2024). Despite these insights, there is not specifically explored the predictive performance of synthetic MRI metrics in assessing neurodevelopmental outcomes related to low-grade GMH-IVH in premature infants. This research gap underscores the necessity for further investigation into the potential of synthetic MRI metrics as predictive indicators in this specific population.

This study aimed to assess the diagnostic efficacy of synthetic MRI conducted at TEA around 38 weeks in forecasting adverse neurodevelopmental outcomes at 18 months among extremely preterm infants with low-grade GMH-IVH. Additionally, it sought to explore whether integrating relaxation times from various brain regions enhances predictive accuracy for neurodevelopmental outcomes.

## 2 Methods

### 2.1 Study participants

This prospective study was conducted at the Third Affiliated Hospital of Zhengzhou University, with written informed consent obtained from each patient’s guardian and approval from the institutional review board of our university for the study design. Prior to MRI data processing, all data were anonymized.

**Figure 1:**
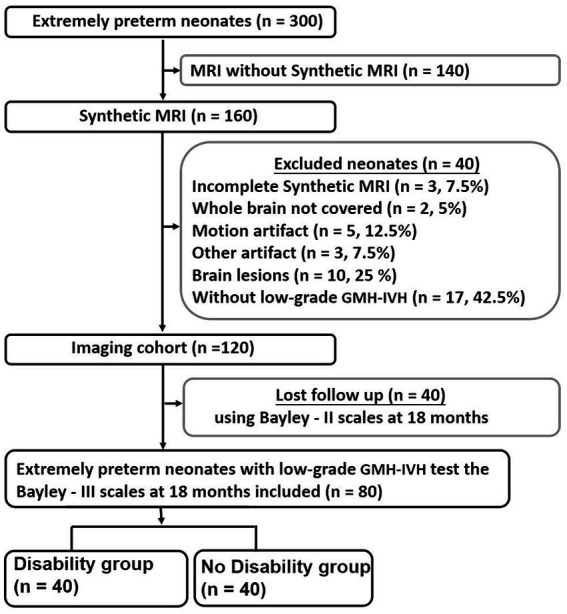
Flowchart shows extremely preterm neonates inclusion and exclusion criteria. Bayley*–*II, Bayley Scales of Infant and Toddler Development 2nd Edition; GMH-IVH, Germinal Matrix Hemorrhage-Intraventricular Hemorrhage.

Extremely preterm neonates admitted to our neonatal intensive care unit between January 2020 and June 2022 were enrolled. Inclusion criteria comprised neonates born (a) at less than 28 weeks of gestational age (GA), (b) with a birth weight (BW) of less than 1,000 g, and (c) who underwent Synthetic MRI around 38 weeks, alongside Bayley–II Scales assessment at 18 months, displaying low-grade GMH-IVH (details provided in Supplementary material S1). Exclusion criteria encompassed neonates with (a) congenital malformations, infections, or metabolic diseases, (b) intracranial hemorrhage graded II or higher on previous ultrasounds, or (c) incomplete Synthetic MRI or any form of artifact. These exclusion criteria were chosen due to their association with poor neurodevelopmental outcomes (Zhang et al., 2021), which could potentially confound the assessment of the significance of quantified MRI values for outcome prediction. A total of 80 extremely preterm infants with low-grade GMH-IVH met the inclusion criteria. The disability group (*n* = 40) was defined by developmental delay, characterized by a score more than 1 standard deviation below the normative mean of 100. The no disability group (*n* = 40) was defined as lacking any of the aforementioned findings, as illustrated in Figure 1.

### 2.2 Magnetic resonance imaging acquisition

Extremely preterm neonates underwent scanning at a corrected GA of 38 weeks ±1 utilizing a 3 T MRI scanner (Pioneer, GE Healthcare, Milwaukee, WI, United States), totaling *n* = 80, fitted with a dedicated 32-channel phased-array head coil. To minimize movement, neonates were positioned on an immobilization pillow, while mini muffs (Natus Medical Inc., San Carlos, CA, United States) were employed to mitigate MRI scanner noise. Throughout the procedure, pulse oximetry and electrocardiographic monitoring ensured neonatal safety.

All extremely preterm infants underwent quantitative synthetic MRI in conjunction with conventional MRI, utilizing a two-dimensional multi-delay multi-echo sequence. This sequence utilized four saturation delays (130, 500, 1,370, and 2,970 ms) and two echoes (22 and 128 ms). Imaging parameters included TR = 4,300 ms, TE = 19.6 ms, slice thickness = 3.0 mm without a gap, field of view = 100 mm × 100 mm, matrix = 288 × 224, number of averages = 1, flip angle = 90 degrees, with an acquisition time of 5 min.

### 2.3 Image processing and analysis

**Figure 2:**
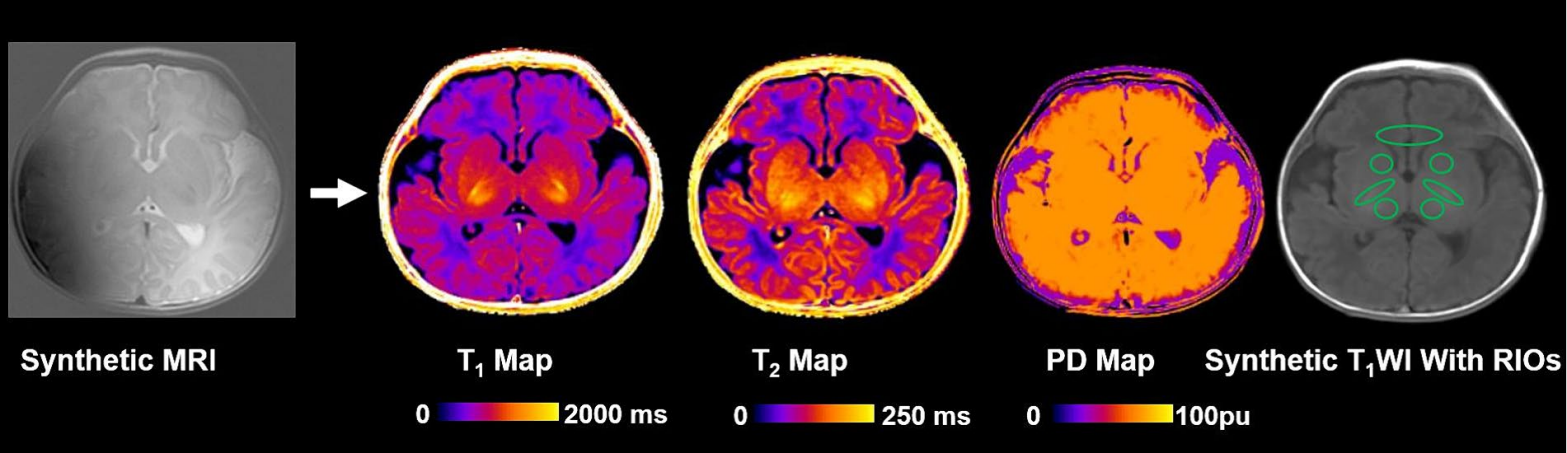
Images show the schematics of the image analysis. T_1_, T_2_ relaxation time and PD maps were generated using synthetic magnetic resonance imaging. PD, Proton density; and ROI, Region of interest.

The image analysis methodology is depicted in Figure 1. A streamlined approach was utilized, where images from a single MRI sequence (Synthetic MRI) were directly fed into the Synthetic MRI software.^1^ Synthetic MRI then automatically generated T1, T2, and proton density maps without the need for extensive pre-processing steps. These maps facilitated the extraction of relaxation times from specific brain regions, including the caudate, thalamus, frontal white matter (FWM), and posterior limb of the internal capsule (PLIC). The locations of intraventricular hemorrhages were defined as lobar, thalamus, basal ganglia, caudate and internal capsules (Eslami et al., 2019). The selection of FWM, thalamus, and caudate as specific regions of interest is justified by their consistent involvement in GMH-IVH (Tortora et al., 2020). Regions of interest (ROIs) were manually drawn by two pediatric radiologists (X.Z. and M.Y.C., both with 10 years of experience) on the following regions (Laule et al., 2007; Zhou et al., 2024): measurements were obtained from both the right and left hemispheres, except for the genu, and then the mean values were used for quantitative analysis. Any disparities were resolved through consensus. The ROI locations are presented in Figure 2. For T_1_, T_2_ relaxation time, and PD maps, ROIs were outlined on synthetic T2-weighted images in Synthetic MRI, automatically generating the relaxation time values.

### 2.4 Neurodevelopmental outcomes

Neurodevelopmental assessments were conducted on neonates at 18 months of corrected age using the second edition of the Bayley Scales of Infant and Toddler Development (Huisenga et al., 2021). A trained examiner with 10 years of experience on the following regions administered the examination, evaluating the Mental Development Index (MDI) and Psychomotor Development Index (PDI). The MDI gauges cognition, focusing on environmental responsiveness, sensory and perceptual abilities, learning, memory, communication, and early language skills. In contrast, the PDI measures motor skills, encompassing both gross and fine motor abilities. The Bayley Scales of Infant and Toddler Development is a widely accepted test, with higher scores indicating better outcomes, and developmental delay defined by a score more than 1 standard deviation below the normative mean of 100.

### 2.5 Statistical analysis

To compare demographic data, socioeconomic status, and neurodevelopmental outcomes between the two groups, we employed the *t*-test or χ^2^ analysis. Group differences in relaxation times were examined using a general linear model, controlling for birth weight and postmenstrual age at MRI potential influencers of relaxation time measurements themselves outcomes (EXPRESS Group, 2010). Interobserver agreement was assessed using the intraclass correlation coefficient (ICC) (details in Supplementary material S2).

Logistic regression analysis was employed to examine the relationship between MRI characteristics and neurodevelopmental outcomes. Variables were adjusted for BW and postmenstrual age at MRI that affect neurodevelopmental outcome (Ling et al., 2013). Receiver operating characteristic (ROC) analysis, along with pairwise comparisons of the area under the ROC curve (AUC), was performed (Hajian-Tilaki, 2013). Internal validation was ensured through bootstrap resampling conducted 1,000 times, and the prediction models were deemed valid if their performance closely matched that of the primary models. Analysis was carried out using SPSS software, version 28.0 (IBM SPSS Statistics), and GraphPad Prism 8 (GraphPad Software, CA, United States). A *p* value <0.05 was considered statistically significant.

## 3 Results

### 3.1 Patients

Table 1 presents a concise overview of demographic, socioeconomic status, and neurodevelopmental outcome data for the subjects. Among the 80 extremely preterm infants with low-grade GMH-IVH, 40 (50%) were classified as having no disability, and 40 (50%) were classified as having a disability. The group with disabilities showed a higher prevalence of bronchopulmonary dysplasia (*p* = 0.013) and lower birth weight (*p* = 0.033) compared to the non-disabled group. MDI in disability group is lower than no disability group (*p* = 0.02). However, no significant differences were observed in the remaining clinical data (*p* > 0.05).

**Table 1:** Demographic data, socioeconomic status, and neurodevelopmental outcomes of all neonates.

	No disability (*N* = 40)	Disability (*N* = 40)	*p*
Demographic			
Gestational age, week	30 (1)	30 (2)	0.744
Postmenstrual age at MR imaging, week	38.3 (1.2)	37.5 (0.3)	0.451
Birth weight, g	1,542 (240.5)	1,433 (145.8)	0.033^*^
Male sex (%)	18 (45)	19 (47.5)	0.437
Multiple birth (%)	8 (20)	9 (22.5)	0.760
Cesarean delivery (%)	32 (80)	35 (87.5)	0.908
Apgar score at 1 min	7 (4)	5.5 (1.5)	0.545
Apgar score at 5 min	6 (4)	8 (2)	0.760
Antenatal corticosteroid use (%)	23 (57.5)	26 (65)	0.754
Postnatal corticosteroid use (%)	24 (60)	26 (65)	0.766
Bronchopulmonary dysplasia (%)	13 (32.5)	22 (55)	0.013^*^
Inotropic support (%)	20 (50)	22 (55)	0.653
Treated patent ductus arteriosus (%)	23 (57.5)	24 (48)	0.673
Total parenteral nutrition (days)	2 (5)	3 (7.5)	0.787
Confirmed postnatal sepsis (%)	5 (12.5)	7 (17.5)	0.176
Necrotizing enterocolitis (%)	6 (15)	5 (12.5)	0.873
Severe retinopathy of prematurity (%)	2 (5)	0	0.323
Socioeconomic status			
Maternal level of education			
Primary/secondary (%)	31 (77.5)	28 (70)	0.865
Under/postgraduate (%)	9 (22.5)	12 (30)	0.913
Paternal level of education			
Primary/secondary (%)	26 (65)	31 (77.5)	0.821
Under/postgraduate (%)	14 (35)	9 (22.3)	0.821
Neurodevelopmental outcome			
MDI	97.432 (6.120)	92.721 (5.332)	0.02^*^
PDI	95.432 (3.239)	95.028 (7.388)	0.477

MDI, Mental development index; PDI, Psychomotor development index.

Data are median (interquartile range) or numbers (%). ^*^p < 0.05.

### 3.2 Measured synthetic MRI values

**Figure 3:**
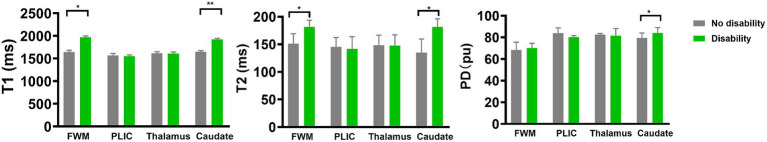
Differences in medians of the relaxation times and PD between extremely preterm infant with no disability and disability groups. Error bars represent interquartile range. In the extremely preterm infant with disability group, T_1_ for FWM and caudate, T_2_ for FWM and caudate, and PD for caudate were higher than no disability group. FWM, Frontal white matter; PLIC, Posterior limb of internal capsule; PD, Proton density. ^*^p < 0.05; ^**^p < 0.001.

Table 2 and Figure 3 illustrate the measured synthetic MRI values following adjusting for BW and postmenstrual age at MRI. The disability group displayed significantly higher T_1_ and T_2_ relaxation times in the FWM (1965.48 ± 34.25 and 181.54 ± 12.29, respectively; *p* < 0.05; FDR *p* value = 0.054 and 0.076, respectively) and caudate (1919.13 ± 25.12 and 172.18 ± 14.60, respectively; *p* < 0.05; FDR *p* value = 0.012 and 0.063, respectively) (Figures 3A,B), as well as PD in the caudate (84.12 ± 4.92, *p* = 0.002; FDR *p* value = 0.012) (Figure 3C), compared to the no disability group, particularly in other selected areas of interests.

**Table 2:** MRI characteristics of the premature infants.

	No disability (*N* = 40)	Disability (*N* = 40)	*p* ^a^	FDR
				*p* value
T_1_ relaxation time, ms				
FWM	1643.51 (36.27)	1965.48 (34.25)	0.013^*^	0.054†
PLIC	1570.75 (41.71)	1550.89 (31.11)	0.650	0.709
Thalamus	1612.73 (35.75)	1610.06 (35.45)	0.402	0.603
Caudate	1644.24 (28.40)	1919.13 (25.12)	<0.001^*^	0.012^†^
T_2_ relaxation time, ms				
FWM	151.23 (17.88)	181.54 (12.29)	0.032^*^	0.076^†^
PLIC	145.376 (21.20)	141.616 (22.10)	0.843	0.843
Thalamus	128.43 (11.30)	137.14 (15.50)	0.068	0.136
Caudate	154.46 (24.70)	172.18 (14.60)	0.021^*^	0.063^†^
Proton density				
FWM	68.46 (7.06)	70.22 (4.32)	0.088	0.150
PLIC	83.97 (4.80)	80.26 (1.50)	0.512	0.652
Thalamus	82.44 (1.26)	81.66 (6.55)	0.544	0.652
Caudate	79.54 (4.53)	84.12 (4.92)	0.002^*^	0.012^†^

PD, Proton density; FWM, Frontal white matter; PLIC, Posterior limb of internal capsule. Data are presented as median (interquartile range). ^a^Adjusted for birth weight and postmenstrual age at MRI. ^*^p < 0.05. ^†^FDR *p* value < 0.1.

### 3.3 Associations with neurodevelopmental outcomes

**Figure 4:**
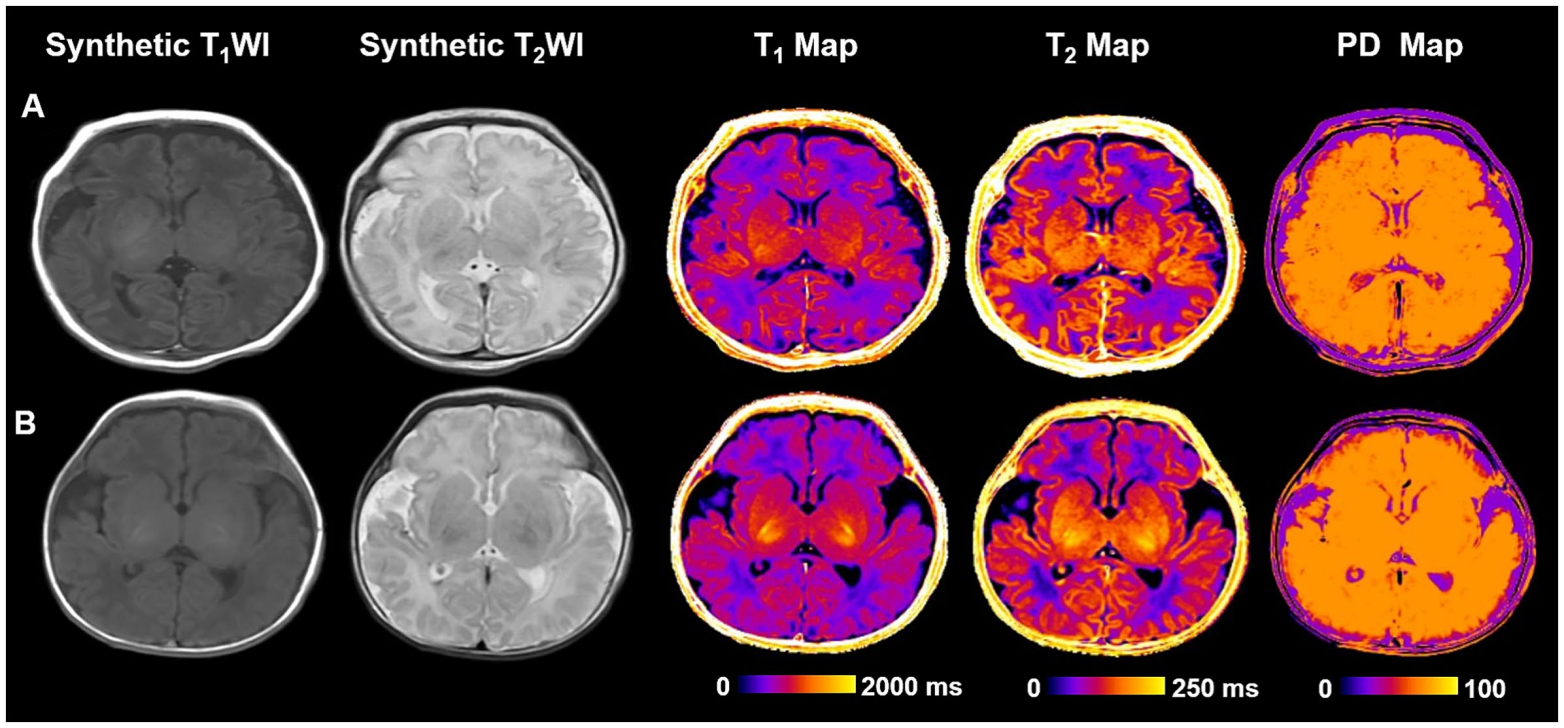
Representative cases of two different neurodevelopmental outcome groups. **(A)** An extremely preterm infant with no disability. An infant weighing 1,630 g born at 32^+5^ weeks had no perinatal risk factors with Bayley–II scores of MDI and PDI at 105 and 99. No definite structural brain abnormalities on T_1_WI and T_2_WI. T_1_, T_2_ relaxation time and PD for PLIC are 1630.4, 133.4, and 81.9 ms, respectively. **(B)** A extremely preterm infant with disability. An infant weighing 1,600 g born at 32^+3^ weeks had no perinatal risk factors with Bayley–II scores of MDI and PDI at 92 and 94. T_2_WI shows bilateral subependymal hemorrhage. T_1_, T_2_ relaxation time and PD for PLIC are 1660.8, 145.6, and 82.7, respectively. Bayley*–*II, Bayley Scales of Infant and Toddler Development 2nd Edition; MDI, Mental development index; PDI, Psychomotor development index; PD, Proton density; and PLIC, Posterior limb of internal capsule.

Table 3 delineates the associations between measured values and neurodevelopmental outcomes. Univariable analysis revealed that T_1_-Caudate and T_2_-Caudate were weakly or significantly associated with MDI scores (*p* < 0.1). After adjusting for birth weight and postmenstrual age at MRI, T_1_-FWM (adj OR, 1.014; 95% CI, 0.961–1.054; *p* = 0.045), T_1_-Caudate (adj OR, 1.039; 95% CI, 1.001–1.053; *p* = 0.032), T2-FWM (adj OR, 1.332; 95% CI, 1.120–1.562; *p* = 0.011), and T_2_-Caudate (adj OR, 1.122; 95% CI, 0.913–1.321; *p* = 0.023) emerged as significant predictors of MDI scores. Figure 4 illustrates representative images of two extremely preterm neonates with relatively high and low MDI scores.

**Table 3:** Relaxation times associated with the MDI score.

Variable	Univariable		Multivariable	
	Unadjusted OR (95% CI)	Unadjusted *p*	Adjusted OR (95% CI)^a^	Adjusted *p*^a^
T_1_ relaxation time, ms				
FWM	0.993 (0.978–1.077)	0.412	1.014 (0.961–1.054)	0.045^*^
Caudate	1.001 (0.932–1.032)	0.055	1.039 (1.001–1.053)	0.032^*^
T_2_ relaxation time, ms				
FWM	0.933 (0.900–0.965)	0.634	1.332 (1.120–1.562)	0.011^*^
Caudate	1.010 (0.981–1.022)	0.051	1.122 (0.913–1.321)	0.023^*^
Proton density				
Caudate	0.921 (0.902–1.113)	0.612	0.985 (0.923–1.108)	0.722

CI, Confidence interval; OR, Odds ratio; FWM, Frontal white matter; PLIC, Posterior limb of internal capsule.

^a^Adjusted for birth weight, and postmenstrual age at MRI. ^*^p < 0.05.

### 3.4 Performance of the relaxation time models

The performance of the prediction models is detailed in Table 4. The standalone T_1_-FWM model demonstrated an AUC of 0.751 and *p* = 0.002. The T1-Caudate model exhibited an AUC of 0.695, and *p* = 0.016. The T2-FWM model displayed an AUC of 0.856 and *p* < 0.001. The T2-Caudate model showcased an AUC of 0.872 and *p* < 0.001. Notably, the combined T_1_-FWM + T_1_-Caudate + T_2_-FWM + T_2_-Caudate model demonstrated an AUC of 0.915 and *p* < 0.001. This amalgamation of information markedly improved overall predictive performance compared to using individual data.

**Table 4:** Diagnostic performance of individual or combined models using relaxation times in different brain regions for predicting adverse neurodevelopmental outcomes.

	AUC	95% CI	*p* value
T_1_-FWM	0.751	0.611–0.843	0.002
T_1_-Caudate	0.695	0.541–0.812	0.016
T_2_-FWM	0.856	0.745–0.947	<0.001
T_2_-Caudate	0.872	0.784–0.961	<0.001
T_1_-FWM + T_1_-Caudate + T_2_-FWM + T_2_-Caudate	0.915	0.896–0.934	<0.001

FWM, Frontal white matter; AUC, Area under the curve.

## 4 Discussion

This study delved into the diagnostic potential of synthetic relaxometry at TEA in predicting adverse neurodevelopmental outcomes among extremely preterm neonates with low-grade GMH-IVH at 18 months of corrected age. The combined T1-FWM + T1-Caudate + T2-FWM + T2-Caudate model demonstrated enhanced predictive power for adverse outcomes, surpassing the efficacy of individual data.

Our findings revealed a notable extension of T1, T2, and/or PD in the FWM and caudate in extremely preterm infants with neurodevelopmental disabilities compared to those without. The prolonged T1 and T2 relaxation times indicated changes in volumetric water content, compartmentalization, and macromolecular microstructures associated with early brain maturation and myelination (Vanderhasselt et al., 2021). This observation aligns with a recent study confirming similar results (Zhang et al., 2021). Hemorrhaging in the FWM leads to myelin breakdown and depletion, altering the myelin structure and resulting in elevated T1 and T2 values (Jara et al., 2022). Caudate involvement in germinal matrix hemorrhage often manifests as prolonged T1, T2, and PD, indicating delayed or impaired myelin development (Schmidbauer et al., 2021). -IVH characteristically initiates in the periventricular germinal matrix. The germinal matrix, located on the head of caudate nucleus and underneath ventricular ependyma, is a highly vascular collection of glial and neuronal precursor cells.

To substantiate the prognosis of neurodevelopmental outcomes, we delved into the MDI and PDI scores of extremely preterm neonates with low-grade GMH-IVH. Meicen et al. similarly identified a heightened risk of cognitive delay in these infants using the Bayley II assessment (Zhou et al., 2024), emphasizing the need for vigilant follow-ups in cases of mild IVH to detect potential developmental delays. However, no intergroup differences in PDI were found in this study. We speculate that the impairment of FWM and caudate in children with cerebral hemorrhage did not affect physical development.

Furthermore, our investigation unveiled elevated T1 and T2 relaxation times in the FWM and caudate among extremely preterm neonates with poorer cognitive scores. A noteworthy negative correlation emerged between T1 and T2 relaxation times in both the FWM and caudate and the MDI score. This aligns with a recent study, where higher T1 and T 2 relaxation times were observed in those with severe postnatal morbidities (Hagiwara et al., 2019). The caudate nucleus is intricately involved in working memory, cognitive function, and emotions. The association between higher T1 relaxation times in the caudate and lower MDI scores suggests a link between delayed myelin development in this region and impaired cognitive function in these neonates (Uccella et al., 2023). Similarly, the frontal lobe, responsible for high-level cognitive functions such as self-control, memory, and emotional expression (Passingham and Lau, 2023), displayed a negative correlation between T1 and T2 relaxation times in the FWM and MDI scores. This correlation implies that delayed myelin development in the FWM during the early neonatal period may negatively impact cognitive functions and vision at 18 months (Zhang et al., 2021; Zhao et al., 2021; Li et al., 2022).

In our study, we found that prolonged T1 and T2 relaxation times in the FWM and caudate serve as robust indicators of adverse neurodevelopmental outcomes. This highlights the vulnerability of these brain regions to cerebral hemorrhage. This affects the development of oligodendrocytes (Riddle et al., 2006). We hypothesize that the extension of relaxation time may signify disrupted cerebral maturation and reactive gliosis associated with low-grade GMH-IVH.

However, our study has several limitations. The relatively small sample size, although justified by the low incidence rate of low-grade GMH-IVH, necessitates cautious interpretation of the findings. Additionally, the neurodevelopmental follow-up was relatively short, confined to a subset of neonates at 18 months of age during the study period. Another constraint is the reliance on a single system for data collection. Future investigations should aim to gather data from diverse systems and time points to enhance both the volume and variety of information.

## 5 Conclusion

Our study illustrates the potential of predicting adverse neurodevelopmental outcomes in extremely preterm infants with low-grade GMH-IVH through synthetic MRI. The combination of the T_1_-FWM + T_1_-Caudate + T_2_-FWM + T_2_-Caudate model significantly improves the prediction of adverse outcomes compared to using individual data. These findings hold promise for assisting clinicians in establishing early intervention strategies in routine clinical practice.

## Data availability statement

The raw data supporting the conclusions of this article will be made available by the authors, without undue reservation.

## Ethics statement

The studies involving humans were approved by Ethics Committee of the Third Affiliated Hospital of Zhengzhou University. The studies were conducted in accordance with the local legislation and institutional requirements. Written informed consent for participation in this study was provided by the participants’ legal guardians/next of kin.

## Author contributions

CZ: Writing – original draft. ZZ: Data curation, Formal Analysis, Writing – original draft. KW: Writing – review & editing. LW: Data curation, Formal Analysis, Writing – original draft. JL: Data curation, Formal Analysis, Writing – original draft. LL: Writing – review & editing. QX: Writing – review & editing. XW: Data curation, Formal Analysis, Writing – original draft. XiaZ: Funding acquisition, Supervision, Writing – review & editing. XinZ: Project administration, Validation, Visualization, Writing – review & editing.

## Footnotes

^1^ https://www.syntheticmr.com/
