# Supplementary material for: Predicting neurodevelopmental outcomes in extremely preterm neonates with low-grade germinal matrix-intraventricular hemorrhage using synthetic MRI
Source: Front Neurosci. 2024 Aug 7;18:1386340. doi: 10.3389/fnins.2024.1386340 (PMC11335622; doi:10.3389/fnins.2024.1386340)
Supplement: Supplementary file 1 [file Data_Sheet_1.docx]

**Supplementary**

**S1** **Diagnostic criteria**

The severity of hemorrhage on ultrasonography is graded based upon the location and extent of the GMH-IVH and presence of lateral ventricular dilatation:

Grade I – Either:

•Bleeding is confined to the germinal matrix (ie, GMH only), or

•GMH plus IVH occupying <10 percent of the lateral ventricular area

Grade II – IVH that occupies 10 to 50 percent of the lateral ventricle area.

Grade III – IVH that occupies >50 percent of the lateral ventricle area and is associated with acute ventricular dilatation.

Periventricular hemorrhagic infarction (PVHI; previously referred to as Grade IV IVH) – Hemorrhagic infarction in periventricular white matter ipsilateral to large IVH.

"Low-grade" GMH-IVH refers to grades I and II.

**S2**

**Table 1 Inter-observer consistency of measurements**

| **Parameters** | **Intraclass correlation coefficient, 95% CI** |
| --- | --- |
| **T_1_ relaxation time** | 0.945(0.856 - 0.961) |
| **T_1_ relaxation time** | 0.914 (0.816 - 0.963) |
| **Proton density** | 0.902 (0.744 - 0.967) |

95% CI = 95% confidence interval.
